# Efficacy and Safety of Coadministered Ezetimibe–Rosuvastatin plus Telmisartan in South Korean Patients with Dyslipidemia and Hypertension: A Multicenter, Randomized, Double-Blind, Active-Controlled, Phase III Trial

**DOI:** 10.3390/jcm12062377

**Published:** 2023-03-19

**Authors:** Zhao-Yan Song, Moo-Hyun Kim, Han-Cheol Lee, Sung-Ji Park, Moo-Yong Rhee, Jong-Il Choi, Sang-Hyun Kim, In-Ho Chae, Young-Joon Hong, Nam-Ho Lee, Gyo-Seung Hwang, Seung-Ho Hur, Jung-Woo Son, Jei-Keon Chae, Hyo-Soo Kim

**Affiliations:** 1 Department of Cardiology, Dong-A University Hospital, Busan 49201, Republic of Korea; sy18846838196@126.com (Z.-Y.S.); kimmh@dau.ac.kr (M.-H.K.); 2 Division of Cardiology, Department of Internal Medicine, Pusan National University Hospital, Busan 49241, Republic of Korea; glaraone@hanmail.net; 3 Division of Cardiology, Department of Medicine, Heart Vascular Stroke Institute, Samsung Medical Center, Sungkyunkwan University School of Medicine, Seoul 06351, Republic of Korea; sungji.park@samsung.com; 4 Cardiovascular Center, Dongguk University Ilsan Hospital, Goyang 10326, Republic of Korea; mooyong_rhee@dumc.or.kr; 5 Division of Cardiology, Department of Internal Medicine, Korea University Anam Hospital, Korea University College of Medicine, Seoul 02841, Republic of Korea; jongilchoi@korea.ac.kr; 6 Division of Cardiology, Department of Internal Medicine, Boramae Medical Center, Seoul National University College of Medicine, Seoul 07061, Republic of Korea; shkimheart@gmail.com; 7 Cardiovascular Center, Seoul National University Bundang Hospital, Seongnam 13620, Republic of Korea; ihchae@snubh.org; 8 Division of Cardiology, Department of Internal Medicine, Chonnam National University Hospital and Medical School, Gwangju 61469, Republic of Korea; hyj200@hanmail.net; 9 Division of Cardiology, Department of Internal Medicine, Kangnam Sacred Heart Hospital, Hallym University College of Medicine, Seoul 07441, Republic of Korea; namholee@hallym.or.kr; 10 Department of Cardiology, Ajou University School of Medicine, Suwon 16499, Republic of Korea; hwanggs@ajou.ac.kr; 11 Division of Cardiology, Keimyung University Dongsan Medical Center, Daegu 42601, Republic of Korea; shur@dsmc.or.kr; 12 Division of Cardiology, Department of Internal Medicine, Yonsei University Wonju College of Medicine, Seoul 26426, Republic of Korea; soneycar@gmail.com; 13 Department of Cardiology, Chunbuk National University Hospital, Jeonju 54907, Republic of Korea; jkchae@jbnu.ac.kr; 14 Division of Cardiology, Department of Internal Medicine, Seoul National University Hospital, Seoul Naional University College of Medicine, Seoul 03080, Republic of Korea

**Keywords:** hypertension, dyslipidemia, ezetimibe, rosuvastatin, telmisartan

## Abstract

Background: The introduction of a fixed-dose combination (FDC) is expected to improve treatment compliance. Methods: There were 181 subjects who were randomized to three groups: ezetimibe–rosuvastatin 10/20 mg + telmisartan 80 mg, ezetimibe–rosuvastatin 10/20 mg, and telmisartan 80 mg. The primary outcomes were change in mean sitting systolic blood pressure (MSSBP) and percentage change in low-density-lipoprotein cholesterol (LDL-C) compared to baseline at week 8. Results: The least-square mean (SE) in MSSBP changes between the ezetimibe–rosuvastatin 10/20 mg + telmisartan 80 mg group and the ezetimibe–rosuvastatin 10/20 mg group were −25.81 (2.34) mmHg and −7.66 (2.45) mmHg. There was a significant difference between the two groups (−18.15 (2.83) mmHg, 95% CI −23.75 to −12.56, *p* < 0.0001). Changes in least-square mean (SE) in LDL-C between the ezetimibe–rosuvastatin 10/20 mg + telmisartan 80 mg group and the telmisartan 80 mg group were −63.82 (2.87)% and −2.48 (3.12)%. A significant difference was observed between the two groups (−61.34 (3.33)%, 95% CI −67.91 to −54.78, *p* < 0.0001). No serious adverse events were observed. Conclusions: Ezetimibe–rosuvastatin plus telmisartan treatment is effective and safe when compared to either ezetimibe–rosuvastatin or telmisartan.

## 1. Introduction

Cardiovascular disease (CVD) has been the primary reason for death for the past 20 years and remains an essential public health issue influencing approximately 200 million people globally, with the burden continuing to increase each year [1,2]. Hypertension and dyslipidemia are the two common risk factors of CVD. According to a survey, over 60% of hypertensive patients had dyslipidemia. Around 50% of patients have dyslipidemia with hypertension [3,4], and their coexistence is known to increase the incidence of CVD events [5]. Therefore, the treatment target should a combination of all factors. However, an addition in the number of tablets can also cause reduction in adherence, which in turn can lead to the failure of therapy [6]. A single fixed-dose combination (FDC) drug can enhance the compliance of patients by decreasing the number of tablets, and at the same time it can effectively maintain the effect of blood pressure (BP) and lipid reduction.

Studies have indicated that the level of blood pressure and cholesterol is positively correlated with CVD, and lowering blood pressure and cholesterol levels can significantly reduce the risk of CVD [7]. Telmisartan is known to have significant protective effects against tissue remodeling in addition to equipotent blood pressure reducing effects compared to other RAS inhibitors [8].

Statins are first-line drugs used to treat dyslipidemia and prevent atherosclerotic cardiovascular disease. Among the statins, rosuvastatin is considered to be the most effective, and large doses of rosuvastatin can reduce LDL-C levels by ≥50% [9]. It is more affordable and generally better tolerated than other statins [10]. However, there are still a large number of subjects who cannot reach the LDL-C goal when treated with statins alone [11]. Moreover, increasing statin dosages can increase the risk of reaction on the hepatic system and muscle. It may therefore be beneficial to combine additional medicines with another mechanisms of action. Ezetimibe can restrain the absorption of cholesterol from the small intestine [12]. Adding ezetimibe to statin treatment, there was an approximately 24% reduction in LDL-C in the IMPROVE-IT study. In addition, combination therapy also reduced the risk of cardiovascular events compared to statins alone [13].

In our study, we sought to evaluate the superiority of ezetimibe–rosuvastatin plus telmisartan versus ezetimibe–rosuvastatin or telmisartan following 8 weeks of treatment in terms of change in MSSBP or percentage change in LDL-C in South Korean patients with both hypertension and dyslipidemia.

## 2. Methods

### 2.1. Study Design

The present study was a multicenter, randomized, three-arm, double-blind, placebo-controlled, phase III trial. Subjects were enrolled from 14 nationwide centers in South Korea. This study conformed with the International Council on Harmonization Good Clinical Practice guidelines, the Declaration of Helsinki and all applicable regulations. Ethics committee approvals were granted by the institutional review board of the participating centers.

After obtaining written informed consent, subjects underwent screening. If subjects met all eligibility criteria, they were required to comply with 4 weeks of therapeutic lifestyle change (TLC) prior to randomization. During the screening period, all lipid-modifying medications and hypertension medications were stopped at least 4 weeks (at least 6 weeks for fibrates) before randomization.

At the baseline visit, subjects were reassessed for eligibility criteria, and if satisfactory, were randomized to three groups: (1) ezetimibe–rosuvastatin 10/20 mg + telmisartan 80 mg (Eze/Ros 10/20 mg + Tel 80 mg), (2) ezetimibe–rosuvastatin 10/20 mg (Eze/Ros 10/20 mg), and (3) telmisartan 80 mg (Tel 80 mg) at a 1:1:1 ratio and with a stratified block randomization method performed by an independent statistician.

To maintain blindness, all subjects were provided with two tablets: the ezetimibe–rosuvastatin 10/20 mg + telmisartan 80 mg group with two active drugs and the ezetimibe–rosuvastatin 10/20 mg group and the telmisartan 80 mg group with one active drug, with one matching placebo. All patients were given two tablets at a fixed time once daily for 8 weeks. All study personnel related to the trial were blinded to the therapy groups.

Subjects were requested to maintain the TLC strictly for the duration of study participation. At day 1 (baseline) and follow-up visits, safety and efficacy evaluations including physical examination, vital signs, laboratory tests, ECG, as well as compliance and adverse events, were evaluated. Follow-up visits were arranged at 4 and 8 weeks.

### 2.2. Study Population 

The inclusion criteria were (a) age ≥ 19 years old (both genders), (b) uncontrolled hypertension indicated by 140 mmHg ≤ MSSBP < 180 mmHg and MSDBP < 110 mmHg (130 mmHg ≤ MSSBP < 180 mmHg in the case of subjects with CKD or diabetes mellitus), and (c) dyslipidemia, defined in accordance with the National Cholesterol Education Program Adult Treatment Panel III (NCEP ATP III) (Supplementary Table S1).

The exclusion criteria were (a) secondary hypertension, secondary dyslipidemia, (b) symptomatic orthostatic hypotension, (c) clinically significant arrythmia, hypertrophic cardiomyopathy, severe obstructive coronary artery disease, aortic stenosis, clinically significant aortic valve or mitral valve stenosis, severe heart failure (NYHA class III and IV), history of ischemic heart disease (myocardial infarction or unstable angina), peripheral vascular disease, severe cerebrovascular disease, undergone percutaneous transluminal coronary angioplasty or coronary artery bypass surgery in the past 6 months, (d) history of myotoxicity following HMG-CoA reductase inhibitor or fibrates, medical or family history of fibromyalgia, myopathy, rhabdomyolysis, or other inherited myopathy, (e) active hepatic disease indicated by consistent elevation in AST or ALT levels with unknown origin or AST, ALT level ≥ 2 times of upper normal limit, (f) creatinine kinase levels ≥ 2 times of upper normal limit, eGFR < 30 mL/min/1.73 m^2^, and (g) any diseases that could influence the results of the study.

### 2.3. Efficacy Variables

The primary outcomes were change in MSSBP in the ezetimibe–rosuvastatin 10/20 mg + telmisartan 80 mg and ezetimibe–rosuvastatin 10/20 mg groups and percentage change in LDL-C in the ezetimibe–rosuvastatin 10/20 mg + telmisartan 80 mg and telmisartan 80 mg groups compared to baseline at week 8.

The secondary outcomes were (1) changes from baseline in MSSBP at weeks 4 and 8 (primary outcomes were excluded), (2) changes from baseline in MSDBP at weeks 4 and 8, (3) percentage changes from baseline in LDL-C at weeks 4 and 8 (primary outcomes were excluded), and (4) percentage changes from baseline in TC, TG, and HDL-C at weeks 4 and 8.

### 2.4. Safety Evaluation

Safety was evaluated according to the adverse events (AEs) checked and documented by the researchers. AEs were categorized into system organ class in accordance with the therapy group and severity and based on the *Medical Dictionary for Regulatory Activities* version 23.0.

### 2.5. Drug Compliance

In our study, the definition of compliance was as follows.

Compliance at each visit (%) = number of days actually taken at each visit/number of days planned at each visit × 100.

Overall compliance (%) = total number of days actually taken/total number of tablets planned × 100.

According to the category of medication compliance (<80%, ≥80%), the frequency and percentage of the subjects are presented.

### 2.6. Statistical Analysis

Efficacy was assessed by using full analysis sets (FAS) as primary analysis, and as a supportive measure, analysis was also conducted on per protocol sets (PPS). Treatment, baseline measurements, and 10-year cardiovascular risk factors at randomization and interaction between treatment and visits were included as a fixed effect in a mixed effect model for repeated measures (MMRM). Unstructured covariance was assumed. Continuous variables were assessed using ANOVA or Kruskal–Wallis test and categorical variables using X2 test or Fisher’s exact test. All analyses were 2-sided, with a 5% significance level, and 95% CI was calculated. SAS version 9.4 (SAS Institute, Inc, Cary, NC, USA) was used for all statistical analyses.

### 2.7. Sample Size

It was hypothesized that the ezetimibe–rosuvastatin 10/20 mg + telmisartan 80 mg group was superior to the ezetimibe–rosuvastatin 10/20 mg and telmisartan 80 mg groups in reducing BP and LDL-C levels. As superiority of both should be met as the co-primary endpoint, no adjustment in multiplicity was done, and each significance level was set at 5%, with 90% power to make the overall power 80%. In a study by Oh et al. [14], the least square (LS) mean between telmisartan 80 mg–rosuvastatin 20 mg (*n* = 80) and rosuvastatin 20 mg (*n* = 40) was −14.4 mmHg (95% confidence interval [CI], −19.8, −9.0). From this result, the treatment difference in SBP between the ezetimibe–rosuvastatin 10/20 mg + telmisartan 80 mg group and the ezetimibe–rosuvastatin 10/20 mg group was assumed to be −9.0 mmHg and the calculated pooled standard deviation (SD) 14.37 mmHg.

The null hypothesis was *H*_0_ = *µ_t_* − *µ_c_* ≥ 0.

*µ_t_*: MSSBP change in the ezetimibe–rosuvastatin 10/20 mg + telmisartan 80 mg group.

*µ_c_*: MSSBP change in the ezetimibe–rosuvastatin 10/20 mg group.

$$\;n=\frac{2{\left(Z_{\alpha }+Z_{\beta }\right)}^{2\;}\sigma^{2}}{{\left(\mu_{t}-\mu_{c}\right)}^{2}}=\frac{2\times {\left(1.96+1.282\right)}^{2\;}\times 14.37^{2}}{{\left(-9.0\right)}^{2}}=53.59\approx 54$$

In the same study, the LS mean between telmisartan 80 mg–rosuvastatin 20 mg (*n* = 80) and telmisartan 80 mg (*n* = 43) was −50.8% (95% CI, −58.1%, −43.5%). From this, the treatment difference in LDL-C level between the ezetimibe–rosuvastatin 10/20 mg + telmisartan 80 mg and the telmisartan 80 mg groups was assumed to be −43.5%, and calculated pooled SD was 19.68%.

The null hypothesis was *H*_0_ = *µ_t_* − *µ_c_* ≥ 0.

*µ_t_*: LDL-C change in the ezetimibe–rosuvastatin 10/20 mg + telmisartan 80 mg group.

*µ_c_*: LDL-C change in the telmisartan 80 mg group.

$$\;n=\frac{2{\left(Z_{\alpha }+Z_{\beta }\right)}^{2\;}\sigma^{2}}{{\left(\mu_{t}-\mu_{c}\right)}^{2}}=\frac{2\times {\left(1.96+1.282\right)}^{2\;}\times 0.1968^{2}}{{\left(0.435\right)}^{2}}=4.3\approx 5$$

We calculated the sample sizes for the two aspects, and 54 subjects per group were chosen in total. A 10% dropout rate was taken into consideration, taking the target number of subjects for each group to 60, resulting in 180 study subjects in total.

## 3. Results

### 3.1. Study Population

**Figure 1:**
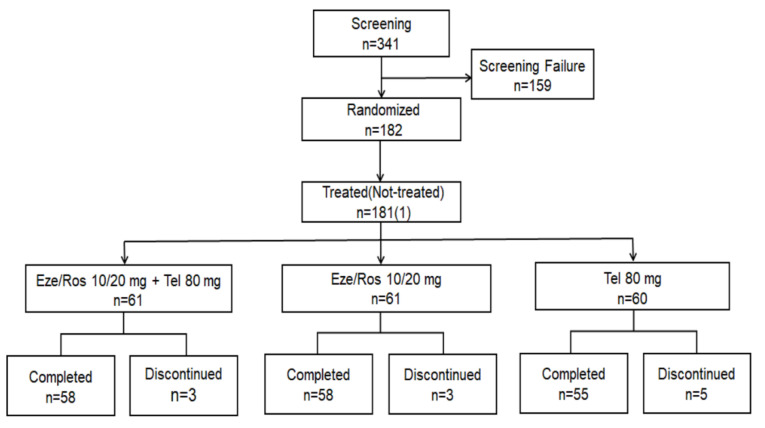
Study flow.

A total of 341 subjects underwent the screening, 182 were eligible for randomization, and 171 completed the 8-week treatment period (as shown in Figure 1). Of the 182 enrolled, 181 were dosed with the study drug at least once (safety set (SS)), FAS included 180 subjects, and 145 subjects were included in the PPS (Supplementary Table S2). No significant differences were observed for baseline characteristics among the three groups (Table 1).

### 3.2. Primary Outcomes

**Figure 2:**
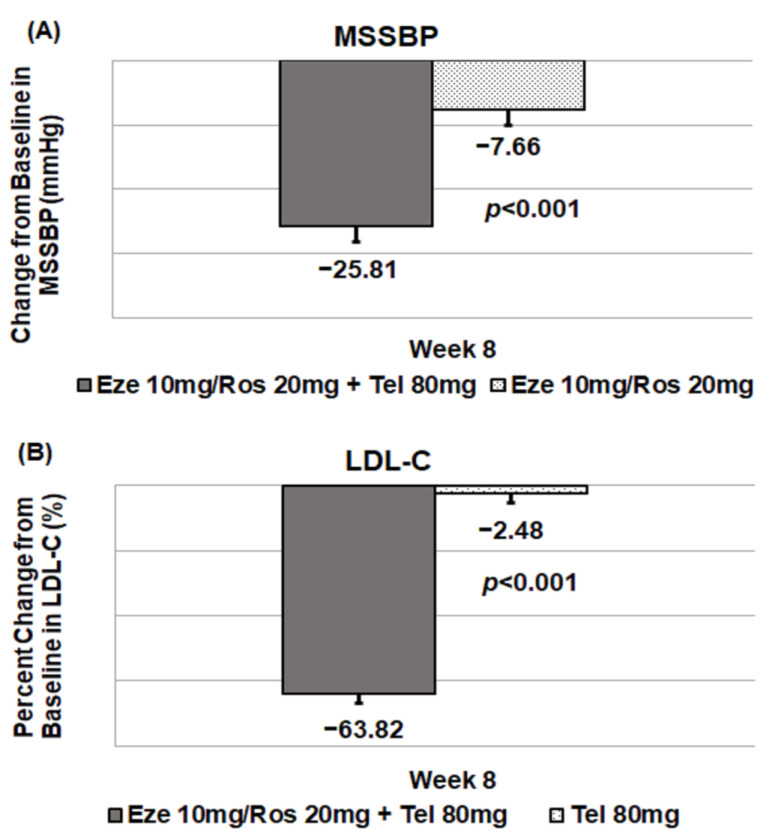
Primary outcomes: change from baseline in MSSBP and LDL-C at week 8. (**A**) MSSBP, (**B**) LDL-C.

The least-square means (SE) in MSSBP changes between the ezetimibe–rosuvastatin 10/20 mg + telmisartan 80 mg group and the ezetimibe–rosuvastatin 10/20 mg group were −25.81 (2.34) mmHg and −7.66 (2.45) mmHg at 8 weeks. There was a significant difference between the two groups in LS means (SE) (95% CI) (−18.15 (2.83) mmHg, −23.75, −12.56, *p* < 0.0001) (Table 2, Figure 2). Changes in LS means for LDL-C between the ezetimibe–rosuvastatin 10/20 mg + telmisartan 80 mg group and the telmisartan 80 mg group were −63.82 (2.87)% and −2.48 (3.12)% at 8 weeks. A significant difference was observed between the two groups in LS means (SE) (95% CI) (−61.34 (3.33)%, −67.91, −54.78, *p* < 0.0001) (Table 2, Figure 2).

### 3.3. Secondary Outcomes

**Figure 3:**
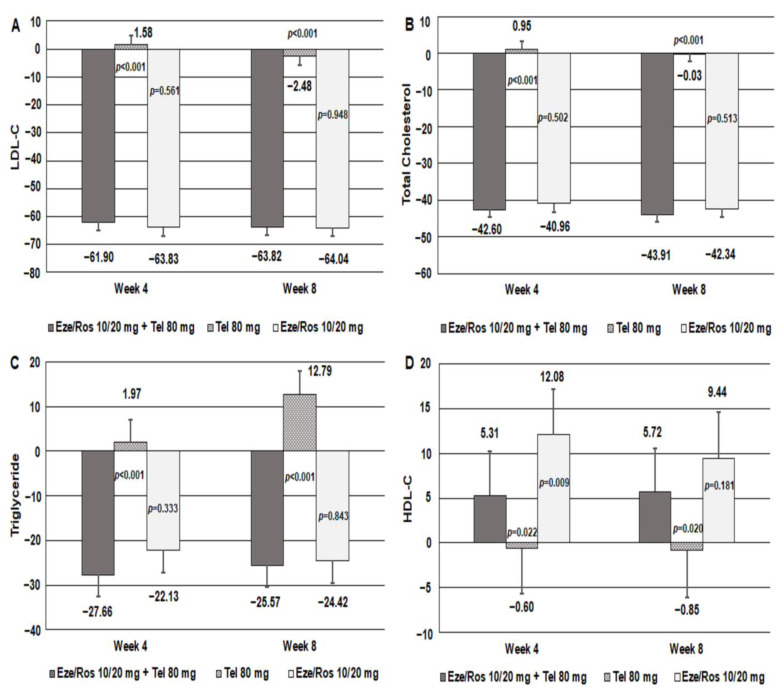
Secondary outcomes at weeks 4 and 8. (**A**) LDL-C, (**B**) Total Cholesterol, (**C**) Triglyceride, (**D**) HDL-C.

The LS means (SE) in MSSBP changes among the ezetimibe–rosuvastatin 10/20 mg + telmisartan 80 mg group, the ezetimibe–rosuvastatin 10/20 mg group, and the telmisartan 80 mg group were −22.93 (2.11) mmHg, −5.85 (2.18) mmHg, and −14.44 (2.20) mmHg at 4 weeks, respectively. There was significant difference between the ezetimibe–rosuvastatin 10/20 mg + telmisartan 80 mg group and the ezetimibe–rosuvastatin 10/20 mg group in LS means (SE) (95% CI) (−17.08 (2.37) mmHg, −21.75, −12.41, *p* < 0.0001). Significant difference was observed between the ezetimibe–rosuvastatin 10/20 mg + telmisartan 80 mg group and the telmisartan 80 mg group in LS means (SE) (95% CI) (−8.49 (2.37) mmHg, −13.17, −3.81, *p* = 0.0004) (Table 2). The LS means (SE) in MSDBP changes among the ezetimibe–rosuvastatin 10/20 mg + telmisartan 80 mg group, the ezetimibe–rosuvastatin 10/20 mg group and the telmisartan 80 mg group were −11.11 (1.24) mmHg, −0.76 (1.28) mmHg and −5.96 (1.28) mmHg at 4 weeks, and −13.20 (1.32) mmHg, −1.17 (1.38) mmHg and −6.20 (1.35) mmHg at 8 weeks, respectively. There was significant difference between the ezetimibe–rosuvastatin 10/20 mg + telmisartan 80 mg group and the ezetimibe–rosuvastatin 10/20 mg group in LS means (SE) (95% CI) at 4 weeks (−10.35 (1.39) mmHg, −13.08, −7.62, *p* < 0.0001) and 8 weeks (−12.03 (1.56) mmHg, −15.10, −8.96, *p* < 0.0001). Significant difference was observed between the ezetimibe–rosuvastatin 10/20 mg + telmisartan 80 mg group and the telmisartan 80 mg group in LS means (SE) (95% CI) at 4 weeks (−5.15 (1.39) mmHg, −7.90, −2.40, *p* = 0.0003) and 8 weeks (−7.00 (1.58) mmHg, −10.12, −3.89, *p* < 0.0001) (Supplementary Table S3). Changes in LS mean (SE) for LDL-C between the ezetimibe–rosuvastatin 10/20 mg + telmisartan 80 mg group and the telmisartan 80 mg group were −61.90 (2.92)% and 1.58 (3.13)% at 4 weeks. Significant difference was observed between the two groups in LS means (SE) (95% CI) −63.48 (3.35)% (−70.09, −56.88, *p* < 0.0001) (Table 2, Figure 3). Compared to the telmisartan 80 mg group, the ezetimibe–rosuvastatin 10/20 mg + telmisartan 80 mg group had statistically significant reduction in total cholesterol. There was significant difference in total cholesterol in LS means (SE) (95% CI) −43.56 (2.46)% (−48.40, −38.71, *p* < 0.0001) at 4 weeks and −43.89 (2.42)% (−48.67, −39.11, *p* < 0.0001) at 8 weeks, respectively (Figure 3). In the ezetimibe–rosuvastatin 10/20 mg + telmisartan 80 mg group, triglyceride levels were decreased at weeks 4 and 8, whereas the telmisartan 80 mg group had triglyceride increased at weeks 4 and 8. Significant difference was observed in triglyceride between the two treatment groups in LS means (SE) (95% CI) −29.63 (5.70)% (−40.89, −18.38, *p* < 0.0001) at 4 weeks, and −38.36 (5.84) (−49.89, −26.83, *p* < 0.0001)% at 8 weeks (Figure 3). In the ezetimibe–rosuvastatin 10/20 mg + telmisartan 80 mg group, HDL-C levels were increased at weeks 4 and 8, whereas the telmisartan 80 mg group had HDL-C decreased at weeks 4 and 8. There was significant difference in HDL-C between the two treatment groups in LS means (SE) (95% CI) 5.91 (2.55)% (0.88, 10.93, *p* = 0.0215) at 4 weeks and 6.57 (2.79) (1.07, 12.07, *p* = 0.0196)% at 8 weeks, respectively (Figure 3).

### 3.4. Safety Assessments

Safety analysis was carried out for subjects who were administered the study drug at least once. TEAEs occurred in a total of 23/181 (12.71%) patients: 9/61 (14.75%) in the ezetimibe–rosuvastatin 10/20 mg + telmisartan 80 mg group, 7/60 (11.67%) in the ezetimibe–rosuvastatin 10/20 mg group, and 7/60 (11.67%) in the telmisartan 80 mg group. No TEAEs regarded as a serious adverse drug reaction (SADR) occurred. Two ADRs led to interruption of the study drug and consisted of supraventricular tachycardia (temporal discontinuation) and ear and neck pain (permanent discontinuation). Both occurred in the telmisartan 80 mg group (Table 3).

### 3.5. Adherence

The mean overall treatment compliance for the three groups was 97.16%, 97.87%, and 96.62%, respectively, indicating strong compliance for all patients (Table 4).

## 4. Discussion

This study sought to evaluate the superiority of ezetimibe–rosuvastatin plus telmisartan versus ezetimibe–rosuvastatin or telmisartan following 8 weeks of treatment in terms of change in MSSBP or percentage change in LDL-C in South Korean patients with both hypertension and dyslipidemia. It was found that (a) the BP-lowering effect of the ezetimibe–rosuvastatin plus telmisartan combination was superior to that of ezetimibe–rosuvastatin and the lipid-modulating effect was superior to telmisartan monotherapy, and (b) in the safety profile, the ezetimibe–rosuvastatin plus telmisartan combination showed outcomes comparable with the other two groups.

Poor patient compliance may have a negative impact on the treatment of hypertension and dyslipidemia. Recent studies have shown the relationship between poor compliance and inadequate control of BP and LDL-C [15,16]. For such patients, reducing the number of pills and improving convenience could improve outcomes [17].

Telmisartan, an angiotensin II receptor blocker, also partially acts on peroxisome proliferator activated receptor-γ(PPAR-γ). In addition to lowering BP, it also has many pleiotropic effects [18]. These extra advantages allow telmisartan to be used to manage patients with multiple cardiovascular risk factors [19].

Rosuvastatin is a member of the statin family. Compared to patients receiving other statins, those treated with rosuvastatin showed statistically significant improvements in LDL-C, total cholesterol and triglyceride [20]. Ezetimibe is a kind of lipid-reducing medicine that can inhibit cholesterol absorption in the diet and bile, and is thought to associate with Niemann–Pick C1-like 1 protein, a key regulator of cholesterol absorption [21]. Due to efficacy and safety considerations, a combination of statins and ezetimibe is the recommended treatment option [22]. Several studies have reported that the proportion of patients using rosuvastatin and ezetimibe to reach the target LDL level is higher than those who increase rosuvastatin alone [23,24].

In a study by Oh et al. [14], in terms of lipid lowering, compared with the Tel 80 mg group, there was significant reduction in LDL-C, total cholesterol, triglyceride and HDL-C in the Tel/Ros 80/20 mg group, and in terms of BP lowering, compared with the Ros 20 mg group, there was significant decrease in MSSBP in the Tel/Ros 80/20 mg group, which was similar to our findings. Jin et al. [25], demonstrated that lipid lowering was significantly greater in the Tel/Aml/Ros 80/5/20 mg group than in the Tel/Aml 80/5 mg group in LDL-C, total cholesterol, triglyceride and HDL-C, while the MSSBP changes exceeded the Tel/Ros 80/20 mg group. This was also in line with our findings.

Our study also showed that the ezetimibe–rosuvastatin plus telmisartan group showed a significant BP reduction compared to the telmisartan group. The effect of rosuvastatin on BP is still controversial because there have been no large-scale, well-designed confirmatory clinical trials demonstrating the antihypertensive effect of rosuvastatin as a primary endpoint in hypertensive patients. Briasoulis et al. [26] conducted a meta-analysis that evaluated data from 40 prospective randomized controlled trials on statin treatment, and found that there was a small but statistically significant decrease in SBP (−2.62 and −3.07 mmHg for patients taking statins and hypertension, respectively). Although further clarification on the mechanism and extent of lowering BP with statin use is needed, it is believed that statins can reduce BP by increasing the bioavailability of nitric oxide and improving arterial compliance [26]. Another possible explanation is due to drug–drug interactions. Pharmacokinetic analysis of the three drugs showed a 16–26% increase in telmisartan concentrations in healthy volunteers [27,28].

For improvements in the lipid spectrum, previous clinical trials of rosuvastatin administration have reported 47−53% reductions in LDL-C [29], and in our study the groups that received ezetimibe–rosuvastatin plus telmisartan and ezetimibe–rosuvastatin showed 63.8% and 64.0% decreases in LDL-C at week 8, respectively. In previous research, patients treated with rosuvastatin reached NCEP ATP III and/or European Society of Cardiology (ESC) target LDL levels ranging from 53% to 94% [30]. In our study, both the ezetimibe–rosuvastatin plus telmisartan and ezetimibe–rosuvastatin groups achieved 95% LDL-C goal attainment at week 8. This confirms that the FDC therapy with ezetimibe–rosuvastatin is effective in LDL-C lowering.

To the best of our knowledge, this is to the first study to evaluate the efficacy and safety of ezetimibe–rosuvastatin plus telmisartan triple fixed dose combination therapy in patients with coexisting hypertension and dyslipidemia. Our findings suggest that the combination therapy provides an effective and convenient therapy choice for patients with both hypertension and dyslipidemia.

### Limitations

This was a phase III licensing study designed to assess the superiority of efficacy and safety of the study drug. Due to its design, the sample was small and the follow-up period short, limiting a more comprehensive evaluation of safety and efficacy. We did not record the education level of patients or their financial situation either. Despite the limitations, the study findings highlight a promising result supporting further development of an FDC, improving therapy options for the management of patients with coexisting hypertension and dyslipidemia. Although this study was not designed to assess a difference in compliance rate between administration of single pills and FDC, the authors assume an FDC will lead to improved treatment compliance in a manner similar to findings in previous studies.

## 5. Conclusions

Compared to either ezetimibe–rosuvastatin FDC or telmisartan monotherapy, ezetimibe–rosuvastatin plus telmisartan triple combination treatment caused a significant reduction in BP and LDL-C without increasing adverse events in South Korean patients with hypertension and dyslipidemia. The introduction of the FDC of these three drugs is expected to enhance compliance and improve clinical outcomes.

## Figures and Tables

**Table 1:** Baseline characteristics of the patients.

	Eze/Ros 10/20 mg + Tel 80 mg (*n* = 60)	Eze/Ros 10/20 mg (*n* = 60)	Tel 80 mg (*n* = 60)	*p*-Value
Age (years)	64.82 ± 10.07	62.92 ± 8.72	65.52 ± 10.63	0.1787
Sex, Male, n (%)	40 (66.67)	40 (66.67)	41 (68.33)	0.9751
Height (cm)	163.20 ± 8.20	163.82 ± 8.54	163.22 ± 8.98	0.9053
Weight (kg)	69.39 ± 11.58	69.83 ± 12.42	69.62 ± 11.72	0.9736
BMI (kg/m^2^)	25.96 ± 3.18	25.91 ± 3.36	25.98 ± 2.70	0.9526
CHD risk factors, n (%)				
Smoking	12 (20.00)	11 (18.33)	7 (11.67)	0.0776
DBP ≥ 90 mmHg	28 (46.67)	30 (50.00)	28 (46.67)	0.9148
HDL-C < 40 mg/dL	19 (31.67)	9 (15.00)	13 (21.67)	0.0907
Family history of premature CHD	4 (6.67)	1 (1.67)	1 (1.67)	0.3702
(−) HDL-C ≥ 60 mg/dL	12 (20.00)	16 (26.67)	12 (20.00)	0.5979
Diabetes mellitus	23 (38.33)	25 (41.67)	18 (30.00)	0.3934
Coronary arterial disease	11 (18.33)	15 (25.00)	9 (15.00)	0.3704
10-y risk > 20%	12 (20.00)	6 (10.00)	7 (11.67)	0.2369
Duration of hypertension (months)	112.03 ± 104.25	108.73 ± 71.22	101.66 ± 83.45	0.6564
Duration of hyperlipidemia (months)	87.74 ± 78.34	83.94 ± 54.20	76.35 ± 56.68	0.7049
Baseline values				
MSSBP (mmHg)	152.41 ± 9.69	154.91 ± 10.83	152.73 ± 9.15	0.3260
MSDBP (mmHg)	88.59 ± 10.59	89.74 ± 9.04	87.85 ± 9.84	0.5693
Pulse (beats/min)	70.62 ± 10.02	70.77 ± 9.49	72.25 ± 12.47	0.6124
Total Cholesterol (mg/dL)	209.45 ± 37.51	217.47 ± 32.27	217.87 ± 31.65	0.2739
Triglyceride (mg/dL)	162.32 ± 67.02	146.17 ± 61.72	158.07 ± 69.49	0.3763
HDL-C (mg/dL)	48.27 ± 12.47	50.97 ± 12.59	49.42 ± 11.57	0.4873
LDL-C (mg/dL)	149.28 ± 30.54	156.12 ± 29.99	156.55 ± 29.82	0.3894
HbA1c (%)	6.21 ± 0.83	6.13 ± 0.70	6.06 ± 0.68	0.7075
Risk group classification, n (%)				
Group 1 (risk factor 0–1)	4 (6.67)	2 (3.33)	3 (5.00)	
Group 2 (risk factor ≥ 2 and 10-year risk ≤ 20%)	8 (13.33)	9 (15.00)	9 (15.00)	
Group 3 (CHD or CHD equivalence or 10-year risk >20%)	46 (76.67)	47 (78.33)	47 (78.33)	

Data are mean values ± SD or n (%). BMI, body mass index; CHD, coronary heart disease; DBP, diastolic blood pressure; Eze, ezetimibe; HDL-C, high-density-lipoprotein cholesterol; LDL-C, low-density-lipoprotein cholesterol; MSDBP, mean sitting diastolic blood pressure; MSSBP, mean sitting systolic blood pressure; Ros, rosuvastatin; Tel, telmisartan.

**Table 2:** Changes from baseline in MSSBP and LDL-C at week 4 and week 8.

	Eze/Ros 10/20 mg + Tel 80 mg (*n* = 60)	Eze/Ros 10/20 mg (*n* = 60)	Tel 80 mg (*n* = 60)
MSSBP (mmHg)			
Baseline	152.41 ± 9.69	154.91 ± 10.83	152.73 ± 9.15
Week 4	129.62 ± 13.22	148.43 ± 14.17	137.98 ± 14.31
MMRM			
LS Means (SE)	−22.93 (2.11)	−5.85 (2.18)	−14.44 (2.20)
LS Mean Difference (SE)		−17.08 (2.37)	−8.49 (2.37)
95%CI		[−21.75, −12.41]	[−13.17, −3.81]
*p*-value		<0.0001	0.0004
Week 8	126.43 ± 15.13	146.53 ± 16.73	136.95 ± 16.81
MMRM			
LS Means (SE)	−25.81 (2.34)	−7.66 (2.45)	−14.78 (2.49)
LS Mean Difference (SE)		−18.15 (2.83)	−11.03 (2.86)
95%CI		[−23.75, −12.56]	[−16.68, −5.38]
*p*-value		<0.0001	0.0002
LDL-C (mg/dL)			
Baseline	149.28 ± 30.54	156.12 ± 29.99	156.55 ± 29.82
Week 4	54.16 ± 30.19	52.57 ± 22.29	153.51 ± 36.12
MMRM			
LS Means (SE)	−61.90 (2.92)	−63.83 (3.07)	1.58 (3.13)
LS Mean Difference (SE)		1.93 (3.32)	−63.48 (3.35)
95%CI		[−4.62, 8.48]	[−70.09, −56.88]
*p*-value		0.5612	<0.0001
Week 8	51.75 ± 23.49	52.62 ± 24.01	149.69 ± 41.34
MMRM			
LS Means (SE)	−63.82 (2.87)	−64.04 (3.07)	−2.48 (3.12)
LS Mean Difference (SE)		0.21 (3.29)	−61.34 (3.33)
95%CI		[−6.28, 6.71]	[−67.91, −54.78]
*p*-value		0.9484	<0.0001

Abbreviations: CI, confidence interval; Eze, ezetimibe; LDL-C, low-density-lipoprotein cholesterol; LS mean (SE), least-square mean (standard error); MMRM, mixed effect model for repeated measures; MSSBP, mean sitting systolic blood pressure; Ros, rosuvastatin; Tel, telmisartan.

**Table 3:** Overall summary of TEAEs.

	Eze/Ros 10/20 mg + Tel 80 mg (*n* = 61)	Eze/Ros 10/20 mg (*n* = 60)	Tel 80 mg (*n* = 60)
Subjects with TEAEs	9 (14.75)	7 (11.67)	7 (11.67)
Subjects with ADRs	1 (1.64)	3 (5.00)	6 (10.00)
Asthenia	0	0	1 (1.67)
Chest pain	0	0	1 (1.67)
Ear pain	0	0	1 (1.67)
Duodenal ulcer	0	0	1 (1.67)
Neck pain	0	0	1 (1.67)
Dizziness	0	0	1 (1.67)
Hematuria	1 (1.64)	0	0
Hypertension	0	1 (1.67)	0
Atrial fibrillation	0	0	1 (1.67)
Supraventricular tachycardia	0	0	1 (1.67)
Alanine aminotransferase increased	1 (1.64)	2 (3.33)	0
Aspartate aminotransferase increased	1 (1.64)	2 (3.33)	0
Gamma-glutamyltransferase increased	1 (1.64)	1 (1.67)	0
Blood alkaline phosphatase increased	1 (1.64)	0	0
Subjects with SAEs	1 (1.64)	0	0
Subjects with Serious ADRs	0	0	0
Subjects with TEAEs Leading to Drug Interruption	0	0	1 (1.67)
Subjects with TEAEs Leading to Drug Withdrawn	0	0	1 (1.67)
Subjects with TEAEs Leading to Death	0	0	0
Subjects with ADRs Leading to Drug Interruption	0	0	1 (1.67)
Subjects with ADRs Leading to Drug Withdrawn	0	0	1 (1.67)
Subjects with ADRs Leading to Death	0	0	0

Abbreviations: ADR, adverse drug reaction; Eze, ezetimibe; Ros, rosuvastatin; SAE, serious adverse event; TEAEs, treatment-emergent adverse events; Tel, telmisartan.

**Table 4:** Treatment compliance.

Variables	Eze/Ros 10/20 mg + Tel 80 mg (*n* = 60)	Eze/Ros 10/20 mg (*n* = 60)	Tel 80 mg (*n* = 60)	Total (*n* = 180)	*p*-Value
Compliance at week 4 (week 0~week 4)	97.50 ± 7.09	97.28 ± 3.59	97.93 ± 4.18	97.56 ± 5.18	0.3059
Subjects with Compliance ≥ 80% at week 4, n (%)	59 (98.33)	59 (98.33)	55 (91.67)	173 (96.11)	NC
Compliance at week 8 (week 4~week 8)	97.20 ± 4.26	98.39 ± 3.08	97.78 ± 4.06	97.79 ± 3.84	0.3701
Subjects with Compliance ≥ 80% at week 8, n (%)	58 (96.67)	58 (96.67)	55 (91.67)	171 (95.00)	NC
Overall Compliance	97.16 ± 4.55	97.87 ± 2.48	96.62 ± 8.21	97.21 ± 5.60	0.4567
Subjects with Compliance ≥ 80%, n (%)	60 (100.00)	60 (100.00)	58 (96.67)	178 (98.89)	0.3296

Abbreviations: Eze, ezetimibe; Ros, rosuvastatin; Tel, telmisartan.

## Data Availability

The data are the property of the authors and can be made available by contacting the corresponding author.

## Supplementary Materials

The following are available at https://www.mdpi.com/article/10.3390/jcm12062377/s1. Table S1. Definition of randomization criteria. Table S2. Overall summary of disposition and analysis set. Table S3. Change from baseline in MSDBP at weeks 4 and 8.
