# Supplementary material for: Efficacy and Safety of Coadministered Ezetimibe–Rosuvastatin plus Telmisartan in South Korean Patients with Dyslipidemia and Hypertension: A Multicenter, Randomized, Double-Blind, Active-Controlled, Phase III Trial
Source: J Clin Med. 2023 Mar 19;12(6):2377. doi: 10.3390/jcm12062377 (PMC10053813; doi:10.3390/jcm12062377)
Supplement: Supplementary file 1 [file jcm-12-02377-s001.zip › jcm-2253850-supplementary.pdf]

Supplementary Information

Table S1. Definition of randomization criteria.

**Hypertension**	140 mmHg ≤ MSSBP <180 mmHg measured at randomization, or 130 mmHg ≤ MSSBP <180 mmHg for patients with diabetes or chronic kidney disease (but only for patients with albuminuria or proteinuria).
**Dyslipidemia**	LDL-C levels and TG criteria measured at the time of randomization corresponding to one of the following criteria according to cardiovascular risk factors:
	1) Cardiovascular Risk Factors[a] ≤1; 160 ≤LDL-C ≤250 mg/dL; TG <400 mg/dL<br>2) Cardiovascular Risk Factors[a] ≥2; 10-year risk[c] <10%; 160 ≤LDL-C ≤ 250 mg/dL; TG < 400 mg/dL<br>3) Cardiovascular Risk Factors[a] ≥2; 10% ≤10-year risk[c] ≤20%; 130 ≤LDL-C ≤ 250 mg/dL; TG < 400 mg/dL<br>4) Coronary artery disease or equivalent[b]; 100 ≤LDL-C ≤250 mg/dL; TG < 400 mg/dL

[a] Current smoking, diastolic blood pressure, DBP ≥90 mmHg, high density lipoprotein cholesterol, HDL-C <40 mg/dL (If HDL-C ≥60 mg/dL, one is subtracted from the total number of risk factors, considered as a protective factor), family history of premature coronary heart disease (< 55 years for first-degree male relatives and < 65 years for female relatives), aged ≥ 45 years old for males or ≥ 55 years old for female

[b] Diabetes: fasting plasma glucose (FPG) ≥126 mg/dL or HbA1c ≥6.5% at screening, or those already diagnosed and receiving treatment for diabetes, or clinically significant coronary and peripheral arterial disease (atherosclerosis, abdominal aortic aneurysm, carotid artery disease, etc.), 10-year risk > 20%

[c] 10-year risk based on Framingham risk score.

Table S2. Overall summary of disposition and analysis set.

	Eze/Ros 10/20 mg + Tel 80 mg	Eze/Ros 10/20 mg	Tel 80 mg	Total
Screening, n				341
Screening Failure, n				159
Randomized, n (%)	61	61	60	182
Treated	61(100.00)	60(98.36)	60(100.00)	181(99.45)
Non-Treated	0	1(1.64)	0	1(0.55)
Status, n (%)				
Completion	58(95.08)	58(95.08)	55(91.67)	171(93.96)
Discontinuation	3(4.92)	3(4.92)	5(8.33)	11(6.04)
Efficacy Sets, n (%)				
Full Analysis Set	60(98.36)	60(98.36)	60(100.00)	180(98.89)
Per-Protocol Set	52(85.25)	48(78.69)	45(75.00)	145(79.67)
Safety Set, n (%)	61(100.00)	60(98.36)	60(100.00)	181(99.45)

Table S3. Change from Baseline in MSDBP at Weeks 4 and 8.

	Eze/Ros 10/20 mg + Tel 80 mg (n=60)	Eze/Ros 10/20 mg (n=60)	Tel 80 mg (n=60)
Baseline	88.59±10.59	89.74±9.04	87.85±9.84
Week 4	76.49±9.33	87.87±11.22	80.97±10.21
MMRM			
LS Means (SE)	-11.11(1.24)	-0.76(1.28)	-5.96(1.28)
LS Mean Difference (SE)		-10.35(1.39)	-5.15(1.39)
95%CI		[-13.08, -7.62]	[-7.90, -2.40]
p-value		<0.0001	0.0003
Week 8	75.35±9.73	87.31±12.63	81.15±9.59
MMRM			
LS Mean (SE)	-13.20(1.32)	-1.17(1.38)	-6.20(1.40)
LS Mean Difference (SE)		-12.03(1.56)	-7.00(1.58)
95%CI		[-15.10, -8.96]	[-10.12, -3.89]
p-value		<0.0001	<0.0001

Abbreviations: CI, Confidence Interval; Eze, Ezetimibe; LS Mean (SE), Least-Square Mean (Standard Error); MMRM, Mixed effect Models for Repeated Measures; MSDBP, Mean Sitting Diastolic Blood Pressure; Ros, Rosuvastatin; Tel, Telmisartan.
